# National School-Based Health Lifestyles Intervention in Chinese Children and Adolescents on Obesity and Hypertension

**DOI:** 10.3389/fped.2021.615283

**Published:** 2021-05-28

**Authors:** Yanhui Dong, Zhiyong Zou, Haijun Wang, Bin Dong, Peijin Hu, Yinghua Ma, Yi Song, Jun Ma

**Affiliations:** ^1^School of Public Health, Institute of Child and Adolescent Health, Peking University, Beijing, China; ^2^Department of Maternal and Child Health, School of Public Health, Peking University, Beijing, China

**Keywords:** obesity, blood pressure, intervention, non-randomized controlled trials, children and adolescents

## Abstract

**Introduction:** This study aimed to examine the effectiveness of the national school-based intervention on both obesity and high blood pressure in Chinese children and adolescents aged 6–18 years.

**Methods:** The national school-based cluster non-randomized controlled trial was done in seven provinces from September 2013 to February 2014. A total of 23,175 children and adolescents in the control group and 25,702 in the intervention group were included in this trial with a mean follow-up of 6.7 ± 0.9 months. Mixed-effects regression models were used to evaluate the effect of the interventions on body weight and blood pressure (BP).

**Results:** A significant upward in the body mass index (BMI) levels but downward in systolic BP (SBP), diastolic BP (DBP), BMI *Z*-scores, SBP Z-scores, and DBP *Z*-scores were witnessed in the intervention group compared to those in the control group (<0.001). Subgroup analyses presented significant intervention effects in children aged 6–12 years for BMI, SBP, DBP, and their standardized values *Z*-scores, but no effective results were found in adolescents aged 13–18 years. Stratification analyses based on the dynamic weight changes presented non-differential HBP, SHBP, and DHBP prevalence gaps between the control and intervention groups. Children aged 6–12 years with higher BMI percentiles at baseline presented obvious declines in SBP and DBP standardized values *Z*-scores.

**Conclusion:** A mean 6-month multi-centered school-based comprehensive obesity intervention in China yields a small to null effect on obesity and hypertension with increasing age; the early age before 12 years may be the key period for interventions, and the younger, the better. Precise and high-intensity interventions targeting the population at different stages of childhood and adolescence are urgently needed to be developed.

**Clinical Trial Registration:** https://www.clinicaltrials.gov/, identifier: NCT02343588

## Introduction

Obesity continues to rise rapidly in many countries (1). In China, the prevalence of overweight and obesity increased from 4.4% in 1995 to 18.4% in 2014 (2). Overweight and obesity commonly begin in childhood or adolescence and predict not only continuing obesity but also increased cardiovascular mortality (3). High blood pressure (HBP) is a further early life indicator of risks of later cardiovascular disease and one that is linked to obesity in childhood (4–6). Thus, theoretically, interventions targeting obesity in childhood may bring double benefits from weight control to healthy blood pressure.

Schools have been regarded as one promising setting for scalable obesity interventions. Since the 1990s, many local school-based intervention studies for obesity prevention have been conducted among Chinese children and adolescents (7, 8). Most trials have targeted the lifestyle of school-aged children including dietary behaviors, physical activity, and sleep duration (9). Some did produce temporary improvements, but without powerful advocating because of the complex and powerful factors that drive the obesity epidemic at the individual, family, school, and societal levels (10). In addition, a few have attempted to shift the school and family environments, key settings for children and adolescents. Additionally, a few have evaluated the effects of health lifestyles on blood pressure (11).

The Health Lifestyles Intervention in Chinese Children and Adolescents (HLI-CCA) was a multicenter cluster non-randomized controlled school-based intervention aiming to prevent obesity in children and adolescents. It included elements of education around nutrition and physical activity as well as modifications to school environments and engagement of families (12). We hypothesized that obesity intervention would have effects not only on obesity but also on the blood pressure in children and adolescents because obesity is becoming one of the leading risk factors for HBP (2). For this reason, we examined whether the school and family interventions for obesity were effective in controlling and preventing HBP in children and adolescents aged 6–18 years.

## Methods

### Study Design

**Figure 1:**
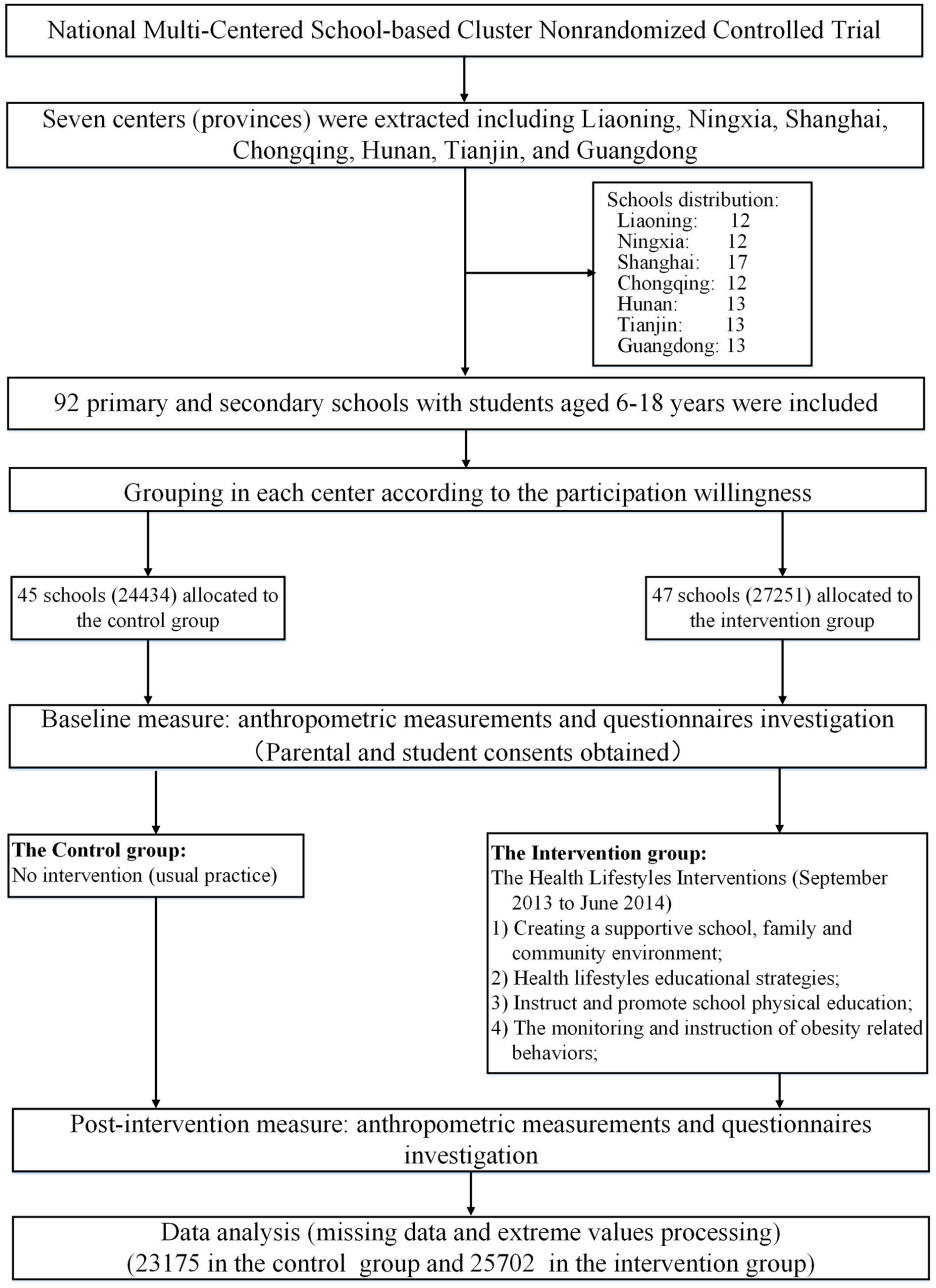
Trial profile and the flow of the participants in the trial.

This national trial with a school-based cluster non-randomized controlled design took place in seven provinces (centers) including Liaoning, Ningxia, Shanghai, Chongqing, Hunan, Tianjin, and Guangdong (Supplementary Figure 1). This study aimed to determine the effectiveness of HLI-CCA in preventing obesity with 6-month intervention duration from September 2013 to February 2014. The flow of the participants and the trial design are presented in Figure 1. The full trial protocol has been published previously in detail (12).

### Sampling and Participants

A multistage cluster non-randomized sampling was used to obtain the representative sample of children and adolescents in each center, where schools meeting the survey requirements, willing to participate, and fulfilling the inclusion criteria were selected according to the trial protocol. In the end, 12–17 primary and secondary schools were recruited in each center. Altogether, 92 schools were included in our study and were assigned to the control or the intervention group. In each school, all participants selected were extracted from *n* classes from each grade (*n* depended on the average size of classes and was no <200 students per school). According to the protocol, the schools and the participants were adjusted slightly to meet the matched standards of balanced schools in the same stratification in each center and the equal distribution of participants in the control or the intervention group (i.e., boy/girl = 1:1, primary school/secondary school = 1:1, urban/rural = 1:1, control/intervention = 1:1).

All participants underwent a regular physical examination every year, and those who had one or more of the following conditions were excluded based on the previous medical history and physical examination data: (1) serious organ disease (e.g., heart, lung, liver, and kidney); (2) abnormal physical development (e.g., pygmyism or gigantism); (3) physical impairment or deformity (e.g., severe scoliosis, pectus carinatum, limp, genu valgum, and genu varum); or (4) acute disease symptoms (e.g., diarrhea and high fever) during the past month and not yet recovered. Furthermore, all principals of the selected schools were assessed comprehensively through a face-to-face interview to ensure the smooth execution of the project.

According to the trial protocol, a planned sample size of 7,000 has 90% power to detect a 10% difference in the obesity intervention group at 5% statistical significance (two-sided) (12). A sample size of 5,000 had 85% power to detect a 10% difference. Ultimately, a total of 5,1685 participants were included in the present analysis with a mean follow-up of 6.7 ± 0.9 months.

### Intervention

The HLI-CCA aimed to deliver a general healthy lifestyle message encouraging a healthy energy balance. In schools assigned to the intervention, the HLI-CCA was delivered to children and adolescents with integrated intervention strategies focused on changing specific behaviors related to energy intake and expenditure, such as decreasing the consumption of sweetened fizzy drinks, increasing the consumption of vegetables, ensuring proper protein intake, reducing sedentary behaviors including screen time, and maintaining at least 1 h of moderate to vigorous physical activity. Integrating the above intervention elements, the comprehensive intervention strategy of the HLI-CCA includes the following four parts during the whole intervention period, which were described in the previously published protocol in detail (12).

**Figure 2:**
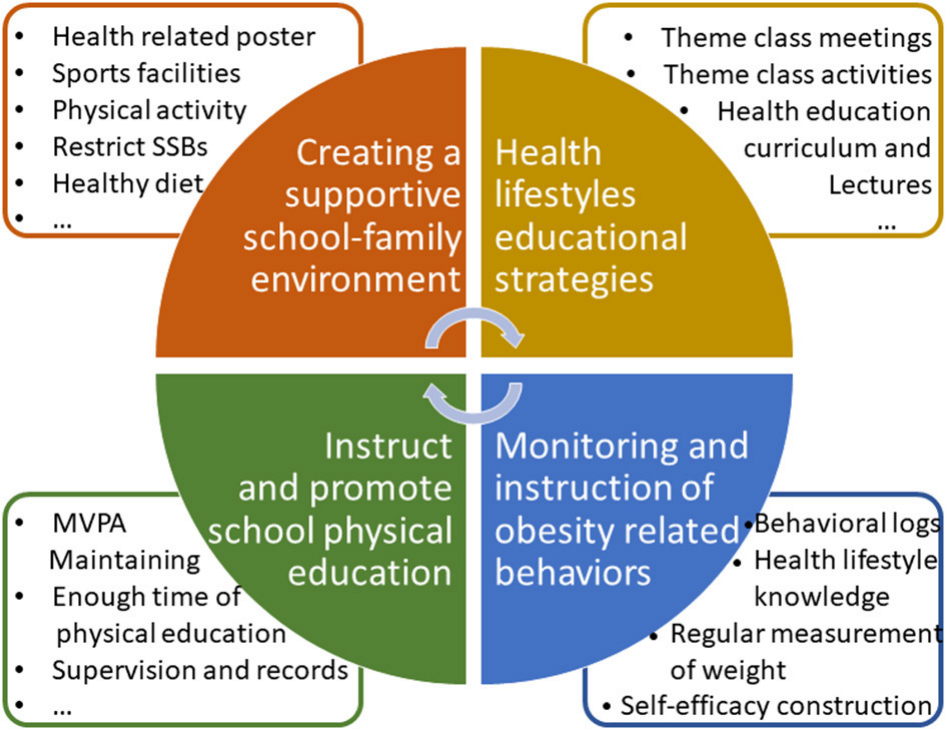
The Health Lifestyles Intervention in Chinese Children and Adolescents (HLI-CCA) intervention model with four strategy designs.

As shown in Figure 2, the intervention model had four strategy designs. Firstly, we targeted the school and family environments through a few strategies with creating a supportive environment of both physical activity and healthier dietary choices. Elements included the provision of the necessary facilities for physical activity in schools, mandating 1-h physical activity time each school day, providing participants and parents with information on obesity prevention through posters, school broadcasts and school website, and restricting sweetened fizzy drinks on the school grounds. Secondly, we developed specialized curriculum and class activities (or campus activities). Intensive health lifestyle educational strategies were established in schools through increasing related knowledge in the health education curriculum, theme class meetings and activities, and health education lectures to parents to enhance the participants' social support in dimensions of peers, schools, and families. Thirdly, strategies were used to instruct and promote school physical education by designing and revising physical education and activities to establish a standardized and rational physical education, such as ensuring enough time for physical education (three times a week) and maintaining moderate to vigorous physical activity 1 h every day under supervision and recording. Fourthly, obesity-related behaviors were monitored and instructed through increasing awareness of self-behaviors using physical activity and dietary behavioral logs, strengthening health lifestyle knowledge with regular measurements of weight and height, and increasing self-efficacy under parental supervision.

Project members, school managers, class teachers, school doctors, parents, and students themselves were all involved in the intervention project. The investigators arranged to supervise the intervention in schools throughout the program. All schools completing the trial would be offered all intervention materials from the project team to reduce loss of follow-up. In the control schools, no specific intervention strategies or activities were carried out throughout the study period, but only records of their daily activities according to ordinary monitoring were conducted by project members. Detailed quality control measures and process evaluation were implemented according to the protocol, including the specialized intervention manual, professional trainings for all the intervention school doctors, health education teachers, and physical education teachers, routine supervision by project managers, and appropriate incentives to avoid loss of follow-up (12).

### Data Collection and Measurement

At baseline and follow-up examinations, information on the demographic characteristics, parental education level, and occupation were collected by questionnaire. All the anthropometric measurements were taken in a sensitive manner in private rooms in both the control and intervention schools. All the measurements followed a standardized procedure by professionals who had passed the training course.

Participants underwent a complete anthropometric measurement at baseline and outcome including height, weight, waist circumference, hip circumference, and blood pressure. About 5% of students would be rechecked, and if the error exceeds 10%, all the students should be measured again. All the participants wore only light clothing and stood erect, barefoot, and at ease while being measured. Height was measured to the nearest 0.1 cm with a portable stadiometer (model TZG, China), and weight was measured to the nearest 0.1 kg with a standardized scale (model RGT-140, China). According to the WHO anthropometric standardization and comparison of different standards, waist circumference and hip circumference were measured to the nearest 0.1 cm, and their measurements were located at 1 cm above the umbilicus and the maximal protrusion of the buttocks, respectively (13, 14).

Blood pressure was measured using an auscultation mercury sphygmomanometer (model XJ1ID, China) with an appropriate cuff for children. Three cuff sizes (7, 9, and 12 cm width) were selected according to the age and upper arm circumference of the children, which stipulated that the cuff bladder width should cover 50–75% of the mid-arm circumference. The mid-upper arm circumference determined the cuff size. The cuff was placed ~2 cm above the crease of the elbow. The child was seated comfortably for at least 10 min prior to the first reading. The feet of children were placed on a platform during BP measurement. Blood pressure was measured twice, with a 1-min break between each measurement. The participants were asked to remain quiet and to sit still while each reading was being taken. Systolic blood pressure was determined by the onset of the first Korotkoff sound (K1) and diastolic blood pressure determined by the fifth Korotkoff sound (K5). The stadiometers, scales, steel tape, and auscultation mercury sphygmomanometer were calibrated before use, and similar instruments were used in all measurements at the investigated schools.

### Outcomes

The two primary outcomes were body mass index (BMI) and blood pressure (BP), including continuous variables [BMI, systolic BP (SBP), diastolic BP (DBP), and their standardized values *Z*-scores] and categorical variables (overweight and obesity, SHBP, DHBP, and HBP). BMI was calculated as body weight (in kilograms) divided by height (in meters) squared. Overweight and obesity were classified using the sex- and age-specific BMI reference values developed by the International Obesity Task Force (IOTF) (15, 16). BMI *Z*-scores were calculated according to the U.S. Centers for Disease Control Growth Charts (www.cdc.gov/growthcharts) (17). BMI values were also grouped according to percentiles for age and sex as follows: less than fifth percentile, fifth to 24th, 25th−49th, 50th−74th, 75th−84th, 85th−94th, and 95th percentile or higher (3). BMI *Z*-scores were also grouped according to their changes during the intervention duration to determine the effects of body weight dynamic changes on the blood pressure as follows: Decline group (BMI *Z*-score changes of −0.5 or less), Stable group (BMI *Z*-score changes between −0.5 and +0.5), and Increase group (BMI *Z*-score changes +0.5 or greater).

Systolic HBP (SHBP) and diastolic HBP (DHBP) were defined as SBP and DBP greater than or equal to the reference age-, sex-, and height-specific 95th centile, respectively, according to the National High Blood Pressure Education Program reference in the Fourth Report. HBP was defined as the SHBP or DHBP of children (18). The SBP and DBP *Z*-scores were calculated according to reference in the Fourth Report.

### Statistical Analysis

Baseline characteristics were described as the mean (SD) for continuous variables or number (percentage) for categorical variables. The analysis of mixed-effects regression models was used to evaluate the effect of intervention on outcomes after adjusting for age, sex, provinces, urban/rural areas, and the baseline disequilibrium for multiple variables. Two-level (individual level and school level) mixed-effects regression models were used in our analyses due to the grouping structure of the data consisting of multiple levels of nested groups. Linear mixed-effects regression models were used to evaluate the intervention on continuous variables (BMI, SBP, DBP, and their standardized values) with β coefficients, whose positive values meant an increase in the variables after intervention, while negative values meant an effective decline with significant *p*-values. Similarly, mixed-effects models for binomial responses were used to evaluate the intervention on categorical variables (overweight and obesity, SHBP, DHBP, and HBP) with odds ratios (ORs), whose values more than 1 meant an increased effect in the variables after intervention, while values <1 meant an effective declined effect with significant *p*-values. Random intercepts with assumed Gaussian distribution were also used in the mixed-effects regression models.

Subgroup analyses were used in our study by age groups as follows: 6–9, 10–12, 13–15, and 16–18 years, which were used to identify the effective or sensitive subgroups for intervention. Allowing for the different intervention effects between the age groups in this study and the age composition of the stages of primary and high schools in China, the study further evaluated the differences in the intervention effects between the two age groups of 6–12 and 13–18 years based on the analysis of the four age groups above. Stratification analyses based on the dynamic change of BMI during the intervention periods were used to assess whether the body weight changes had mediating effects on the intervention effects by age groups. Stratification analyses based on BMI percentiles at baseline were used to explore which groups with different initial body weights were sensitive to intervention. Due to the blood pressure sustained increment along with age by genders, we used the blood pressure standardized values to assess the highly sensitive population with weight at baseline. In each BMI percentile group, changed values of variables = (Standardized value Z-scores in post-intervention in intervention group—Standardized value Z-scores at baseline in the intervention group)—(Standardized value Z-scores in post-intervention in the control group—Standardized value Z-scores at baseline in the control group). The same analyses of the mixed-effects regression models were conducted for the different BMI percentile groups with β coefficients and ORs to further quantify the intervention effects of BMI on blood pressure. Statistical analyses were conducted using Stata version 14.0 (StataCorp). Statistical significance was defined using a two-sided test with *p*-values of 0.05.

## Results

### Participants

The baseline characteristics of the study participants are shown in Table 1. The control and intervention groups consisted of 23,175 and 25,702 participants, respectively, with approximately half being males. The mean age at the baseline medical evaluation was 10.9 ± 3.2 and 11.3 ± 3.3 years in the control and intervention groups, respectively. A significant difference between groups was found for parental educational level, parental occupation, and monthly household income, but not in being from a one-child family. The control group had lower height, weight, BMI, hip circumference, waist circumference, and lower prevalence of HBP and SHBP than those in the intervention group. There is no difference in the prevalence of overweight and obesity and DHBP or the levels of SBP and DBP between the two groups.

**Table 1:** Characteristics of the participants at baseline.

**Characteristics**	**Control**	**Intervention**	***p*-value**
*N*	23,175	25,702	
Boys (%)	50.7	50.4	0.553
Age (years)	10.9 (3.2)	11.3 (3.3)	<0.001
Socio-demographics, n (%)
Only child	15,140 (65.3)	16,984 (66.1)	0.081
Paternal educational level
Father senior high school or above	12,200 (53.7)	14,881 (59.5)	<0.001
Mather senior high school or above	11,247 (49.6)	13,902 (55.6)	<0.001
Father occupation			<0.001
Commerce and services	6,055 (26.1)	6,985 (27.2)	
Professional and technical	6,668 (28.8)	7,073 (27.5)	
Administrator and clerk	2,191 (9.5)	2,986 (11.6)	
Other	8,261 (35.6)	8,658 (33.7)	
Maternal occupation			<0.001
Commerce and services	6,699 (28.9)	7,616 (29.6)	
Professional and technical	4,229 (18.2)	4,610 (17.9)	
Administrator and clerk	1,214 (5.2)	1,819 (7.1)	
Other	11,033 (47.6)	11,657 (45.4)	
Monthly household income (RMB)			<0.001
<2,000	2,136 (9.2)	2,062 (8.0)	
2,000–5,000	5,846 (25.2)	6,219 (24.2)	
5,000–8,000	4,021 (17.4)	4,310 (16.8)	
More than 8,000 or refused to answer	11,172 (48.2)	13,111 (51.0)	
Anthropometry and prevalence, n (%)/mean (SD)
Height, mean (SD) (cm)	143.8 (16.6)	145.9 (17.0)	<0.001
weight, mean (SD) (kg)	39.6 (15.3)	40.9 (15.4)	<0.001
BMI, mean (SD) (kg/m^2^)	18.4 (3.8)	18.5 (3.7)	0.002
HC, mean (SD) (cm)	75.8 (11.9)	77.1 (12.0)	<0.001
WC, mean (SD) (cm)	64.2 (10.7)	64.8 (10.8)	<0.001
Overweight and obesity, *n* (%)	4,966 (21.4)	5,377 (20.9)	0.170
SBP, mean (SD) (mmHg)	105.1 (11.6)	105.3 (11.8)	0.070
DBP, mean (SD) (mmHg)	66.9 (8.3)	66.9 (8.5)	0.762
SHBP, *n* (%)	1,475 (6.4)	1,449 (5.7)	0.001
DHBP, *n* (%)	1,273 (5.5)	1,329 (5.2)	0.116
HBP, *n* (%)	2,181 (9.4)	2,203 (8.6)	0.001

*BMI, body mass index; HC, hip circumference; SBP, systolic blood pressure; DBP, diastolic blood pressure; WC, waist circumference; SHBP, systolic high blood pressure; DHBP, diastolic high blood pressure; HBP, high blood pressure*.

### Overall Intervention Effect on Weight and Blood Pressure

After an average 6 months' intervention with participation rates of 94.3% in the intervention group and 94.8% in the control group, the intervention group had an increase of BMI values of 0.14 kg/m^2^ compared to 0.11 kg/m^2^ in the control group. The β coefficient of 0.04 (*p* <0.001) indicated a greater increase in BMI in the intervention group. Both control and intervention groups had declines in the prevalence of overweight and obesity, but with a non-significant trend for lower levels in the intervention effects (OR = 0.85, 95% CI = 0.70–1.05, *P* = 0.134) after adjusting for age, sex, provinces, urban/rural areas, and the baseline disequilibrium for multiple variables. As for the BMI *Z*-scores, the negative significant β coefficient of −0.0035 (*p* = 0.002) indicated that there was a significant decrease of the BMI *Z*-scores in the intervention group compared to the control group.

An overall larger decrease of the SBP (−0.46 mmHg) and DBP (−0.88 mmHg) levels was found in the intervention compared with the control group (0.33 and −0.41 mmHg). The negative significant β coefficients, after adjusting for age, sex, provinces, urban/rural areas, and the baseline disequilibrium for multiple variables, predicted that the intervention would lead to a 0.77 and a 0.46-mmHg absolute decrease in SBP and DBP, as well as to a 0.047 and a 0.033 decrease in the SBP and DBP *Z*-scores for children and adolescents aged 6–18 years, respectively (*p* <0.001). Similar results were found in both sexes, with 0.78 mmHg (SBP) and 0.40 mmHg (DBP) absolute decreases in boys and 0.75 and 0.53 mmHg absolute decreases in girls, respectively. However, overall, no significant difference for the intervention effects existed for HBP, SHBP, and DHBP, except for DHBP in girls, although we saw a larger decline in the prevalence of HBP, SHBP, and DHBP in the intervention group than that in the control group. For example, the prevalence of HBP decreased from 8.6 to 5.8% with −2.8 percentage points, which was higher than that in the control group (−2.6 percentage points) with non-statistical effects of the OR value (0.92, 95% CI = 0.83–1.02, *p* = 0.107) (Table 2).

**Table 2:** Intervention effect on child body mass index (BMI), systolic blood pressure (SBP), diastolic blood pressure (DBP), and their *Z*-scores, as well as overweight and obesity, SHBP, DHBP, and HBP in both sexes.

**Indicators**	**Time**	**Boys**		**Girls**		**Total**	
		**Control**	**Intervention**	**Control**	**Intervention**	**Control**	**Intervention**
BMI (kg/m^2^)	Baseline, mean (SD)	18.75 (3.91)	18.83 (3.90)	18.11 (3.58)	18.25 (3.49)	18.44 (3.76)	18.54 (3.72)
	Post-intervention, mean (SD)	18.79 (3.98)	18.89 (3.97)	18.29 (3.71)	18.48 (3.63)	18.54 (3.86)	18.68 (3.81)
	Change	0.04	0.06	0.18	0.22	0.11	0.14
	Effect for β coefficients^a^	0.02 (0.00, 0.05)		**0.05 (0.03, 0.08)**		**0.04 (0.02, 0.05)**	
	*p*-values	0.054		** <0.001**		** <0.001**	
SBP (mmHg)	Baseline, mean (SD)	106.31 (11.93)	106.63 (12.29)	103.79 (11.15)	103.86 (11.14)	105.06 (11.62)	105.26 (11.82)
	Post-intervention, mean (SD)	106.49 (11.91)	106.01 (12.52)	104.26 (10.74)	103.55 (11.20)	105.39 (11.40)	104.79 (11.95)
	Change	0.19	−0.62	0.47	−0.3	0.33	−0.46
	Effect for β coefficients^a^	**−0.78 (−1.09**, **−0.47)**		**−0.75(−1.06**, **−0.44)**		**−0.77 (−0.99**, **−0.55)**	
	*p*-values	** <0.001**		** <0.001**		** <0.001**	
DBP (mmHg)	Baseline, mean (SD)	67.42 (8.55)	67.36 (8.66)	66.44 (8.06)	66.46 (8.36)	66.94 (8.33)	66.91 (8.53)
	Post-intervention, mean (SD)	66.98 (8.39)	66.51 (8.82)	66.07 (7.83)	65.55 (8.15)	66.53 (8.13)	66.03 (8.51)
	Change	−0.44	−0.86	−0.37	−0.91	−0.41	−0.88
	Effect for β coefficients^a^	**−0.40 (−0.66**, **−0.15)**		**−0.53 (−0.77**, **−0.28)**		**−0.46 (−0.64**, **−0.29)**	
	*p*-values	**0.002**		** <0.001**		** <0.001**	
BMI *Z*-scores	Baseline	0.443 (1.380)	0.383 (1.390)	0.039 (1.129)	0.009 (1.131)	0.244 (1.279)	0.197 (1.281)
	Post-intervention	0.294 (1.377)	0.246 (1.368)	−0.036 (1.135)	−0.044 (1.117)	0.131 (1.274)	0.102 (1.258)
	Change	−0.149	−0.137	−0.075	−0.053	−0.113	−0.095
	Effect for β^a^	**−0.043 (0.017)**		−0.027 (0.015)		**−0.035 (0.011)**	
	*p*-values	**0.014**		0.065		**0.002**	
SBP *Z*-scores	Baseline	0.129 (0.972)	0.081 (0.972)	0.014 (0.979)	−0.044 (0.974)	0.072 (0.978)	0.019 (0.975)
	Post-intervention	0.042 (0.958)	−0.081 (0.968)	−0.029 (0.935)	−0.154 (0.954)	0.007 (0.948)	−0.117 (0.962)
	Change	−0.087	−0.162	−0.043	−0.11	−0.065	−0.136
	Effect for β^a^	**−0.044 (0.012)**		**−0.050 (0.012)**		**−0.047 (0.009)**	
	*p*-values	** <0.001**		** <0.001**		** <0.001**	
DBP *Z*-scores	Baseline	0.512 (0.681)	0.479 (0.687)	0.443 (0.700)	0.409 (0.726)	0.478 (0.691)	0.444 (0.707)
	Post-intervention	0.418 (0.665)	0.348 (0.689)	0.360 (0.675)	0.280 (0.697)	0.389 (0.670)	0.314 (0.694)
	Change	−0.094	−0.131	−0.083	−0.129	−0.089	−0.13
	Effect for β^a^	−0.038 (0.009)		−0.028 (0.009)		−0.033 (0.006)	
	*p*-values	<0.001		<0.001		<0.001	
OWOB	Baseline (%)	26.4	25.9	16.3	15.9	21.4	20.9
	Post-intervention (%)	23.8	22.9	15.2	14.7	19.6	18.9
	Change (%)	−2.6	−2.9	−1.1	−1.2	−1.9	−2.1
	Effect for OR^a^	0.90 (0.76, 1.07)		0.97 (0.74, 1.26)		0.85 (0.70, 1.05)	
	*p*-values	0.246		0.798		0.134	
SHBP	Baseline (%)	7.2	6.3	5.6	5	6.4	5.7
	Post-intervention (%)	5.1	4.1	3.7	3.1	4.4	3.7
	Change (%)	−2.1	−2.1	−1.8	−1.9	−2	−2
	Effect for OR^a^	0.94 (0.79, 1.12)		0.91 (0.75, 1.10)		0.93 (0.82, 1.05)	
	*p*-values	0.502		0.332		0.245	
DHBP	Baseline (%)	5.5	4.8	5.5	5.6	5.5	5.2
	Post-intervention (%)	4	3.3	3.9	3.2	4	3.3
	Change (%)	−1.5	−1.5	−1.6	−2.4	−1.5	−1.9
	Effect for OR^a^	0.97 (0.81, 1.16)		**0.78 (0.65, 0.94)**		**0.87 (0.76, 0.99)**	
	*p*-values	0.744		**0.009**		**0.033**	
HBP	Baseline (%)	10.1	9	8.8	8.2	9.4	8.6
	Post-intervention (%)	7.4	6.2	6.3	5.3	6.8	5.8
	Change (%)	−2.7	−2.8	−2.5	−2.9	−2.6	−2.8
	Effect for OR^a^	0.95 (0.82, 1.09)		0.89 (0.76, 1.03)		0.92 (0.83, 1.02)	
	*p*-values	0.466		0.121		0.107	

a *effects of interventions for BMI, systolic BP, diastolic BP, BMI Z-score, systolic BP and diastolic BP Z-scores, overweight and obesity, systolic HBP, diastolic HBP, and HBP using the mixed-effects regression models adjusting for age, sex (total), provinces, urban/rural areas, and the baseline disequilibrium for socio-demographic indicators*.

*The bold values indicated the statistically significant values for the effect for β and OR with p <0.05*.

### Subgroup Analysis by Age

**Figure 3:**
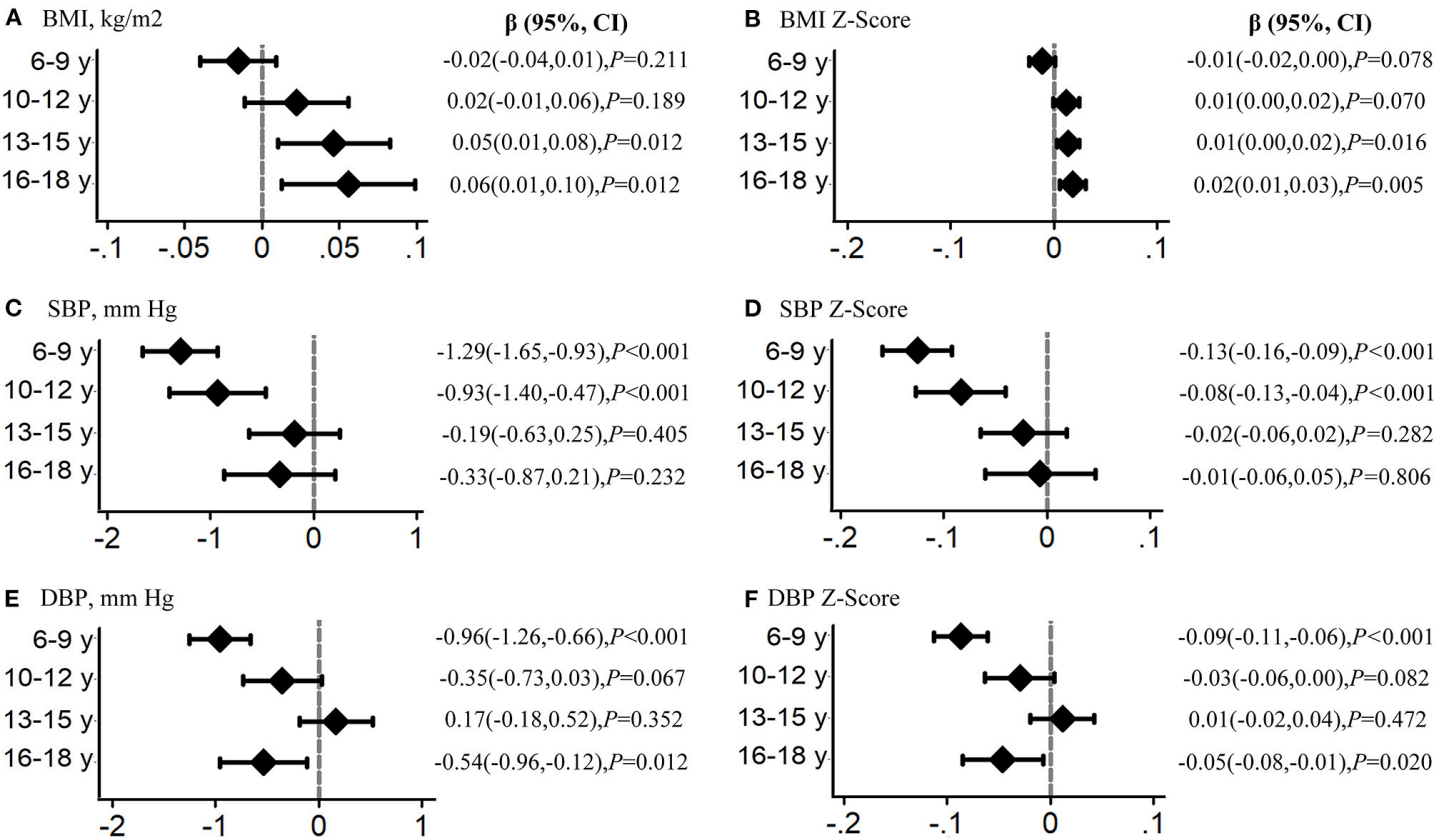
Analysis by age group of the intervention effects on child body mass index (BMI), systolic blood pressure (SBP), and diastolic blood pressure (DBP). β values indicate the effects of interventions for BMI, systolic BP, and diastolic BP using the mixed-effects regression models in each age group adjusting for age, sex (total), and the baseline disequilibrium for socio-demographic indicators. The subfigure **(A)** reflect the intervention effect in BMI, **(B)** for BMI *Z*-Score, **(C)** for systolic blood pressure, **(D)** for systolic blood pressure *Z*-Score, **(E)** for diastolic blood pressure, and **(F)** for diastolic blood pressure *Z*-Score.

**Figure 4:**
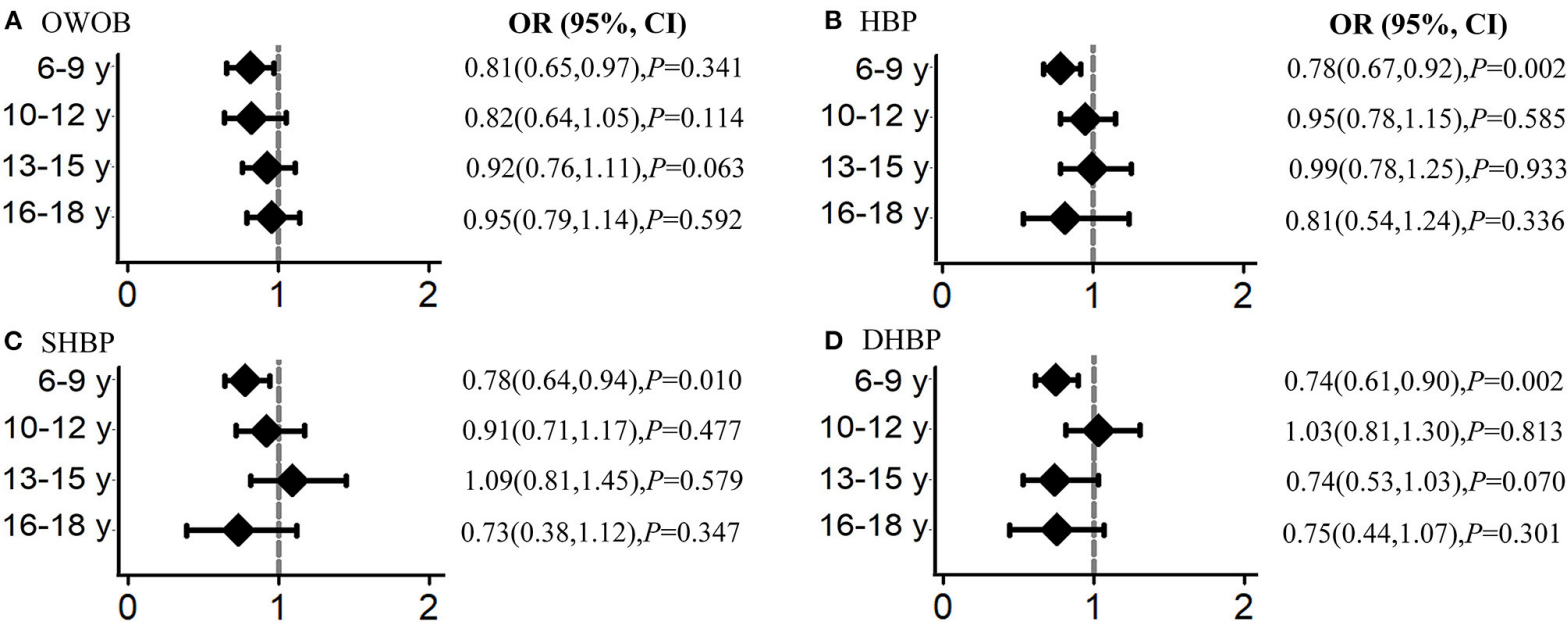
Analysis by age group of the intervention effects on overweight and obesity (OWOB), systolic high blood pressure (SHBP), diastolic high blood pressure (DHBP), and high blood pressure (HBP). Odds ratios (ORs) indicate the effect of interventions for BMI, systolic BP, and diastolic BP using the mixed-effects regression models in each age group adjusting for age, sex (total), and the baseline disequilibrium for socio-demographic indicators. The subfigure **(A)** reflect the intervention effect in overweight and obesity, **(B)** for high blood pressure, **(C)** for systolic high blood pressure, **(D)** for diastolic high blood pressure.

We further divided the participants into four age groups to evaluate the intervention effect on weight and blood pressure, as follows: 6–9, 10–12, 13–15, and 16–18 years. Overall significant intervention effects were found in children aged 6–12 years for BMI, SBP, DBP, and their standardized value *Z*-scores, with statistically significant β coefficients, particularly in those aged 6–9 years with relatively better intervention effects. However, there seemed no obvious intervention effects for adolescents aged 13–18 years (Figure 3). Similar results were found for the prevalence of overweight and obesity, HBP, SHBP, and DHBP, with significant ORs in children aged 6–12 years, but null ORs in adolescents aged 13–18 years (Figure 4). For example, the intervention resulted in 0.03 kg/m^2^ in BMI, 1.31 mmHg in SBP, and 0.98 mmHg absolute decreases and in 19% in overweight and obesity, 22% in HBP, 22% in SHBP, and 26% in DHBP reduced risks in children aged 6–9 years after adjusting for several confounders and the baseline disequilibrium for several indicators.

### Influence of Weight Change on Blood Pressure

**Figure 5:**
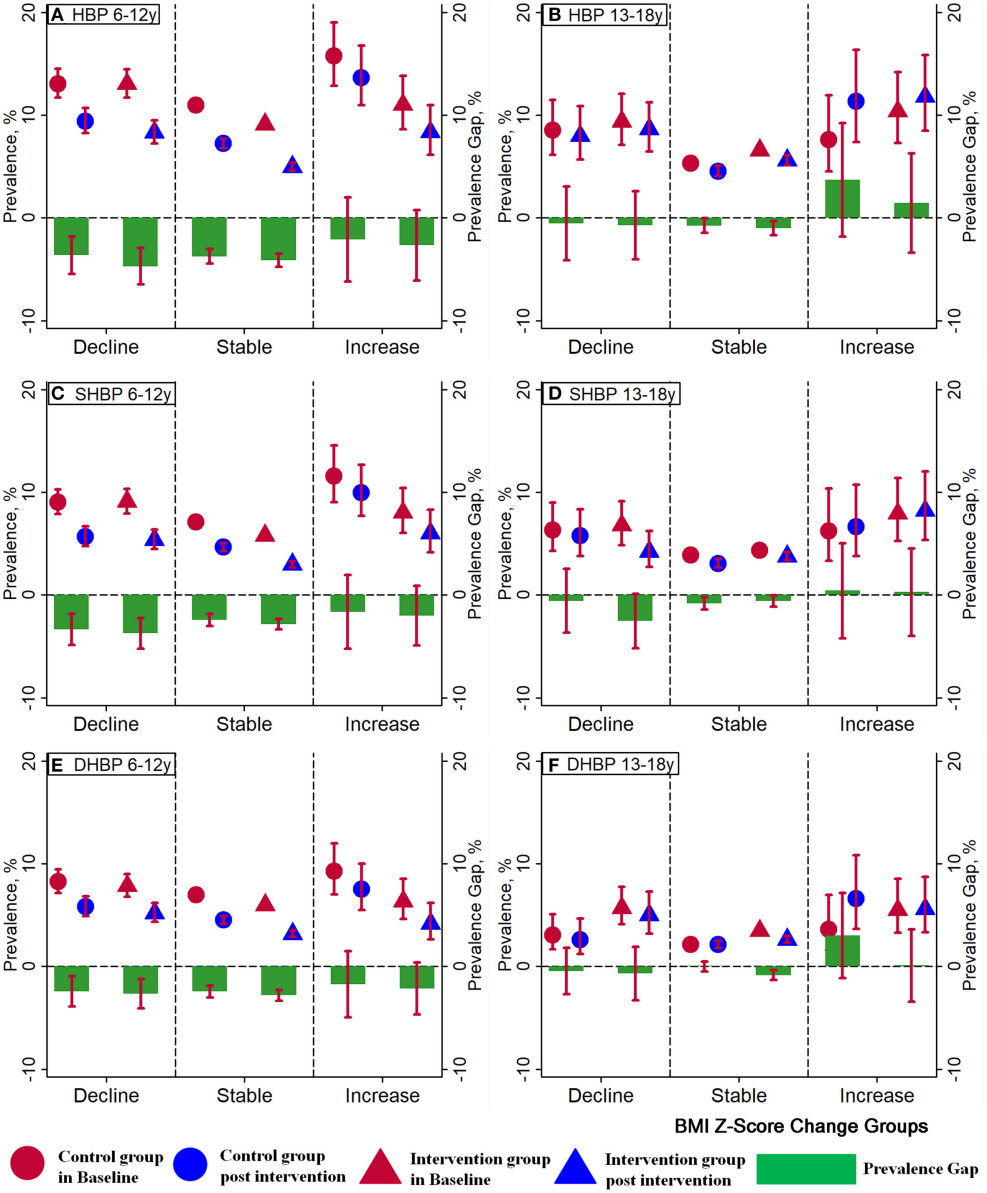
Effect of interventions on high blood pressure (HBP, A for 6-12 years, B for 13-18 years), systolic high blood pressure (SHBP, C for 6-12 years, D for 13-18 years), and diastolic high blood pressure (DHBP, E for 6-12 years, F for 13-18 years) in different BMI *Z*-score change groups. Three BMI *Z*-score change groups were defined for the BMI *Z*-score changes between baseline and post-intervention: BMI *Z*-score changes between −0.5 and 0.5 as the Stable group, < −0.5 as the Decline group, and more than 0.5 as the Increase group. *Red circles* represent the prevalence of HBP, SHBP, and DHBP in the control group at baseline and *blue circles* represent that after the intervention. The prevalence of HBP, SHBP, and DHBP in the intervention group at baseline is represented by *red triangles* and that after the intervention is represented by *blue triangles*. *Green bars* represent the prevalence gaps of HBP, SHBP, and DHBP between the baseline and post-intervention.

Even though the intervention group in both age groups presented larger decrements in the HBP, SHBP, and DHBP prevalence than those in the control group in each body weight change group, a non-statistically significant difference existed between the control group and the intervention group for prevalence gap with overlapped 95% confidence interval (Figure 5). When further stratified by the BMI percentile categories at baseline, children aged 6–12 years presented more obvious declines in the SBP and DBP standardized value *Z*-scores in all BMI percentile groups compared to adolescents aged 13–18 years. In group of 6–12-year-olds, there were reductions in the prevalence of HBP, SHBP, and DHBP, even in subjects who increased their BMI *Z*-scores, while in the group of 13–18-year-olds, the increases in the BMI scores were associated with increases in the prevalence of HBP, SHBP, and DHBP. Furthermore, children aged 6–12 years with higher BMI percentiles had larger decrements in the SBP and DBP *Z*-scores than their peers with lower BMI percentiles (Supplementary Figure 2).

### Adverse Events Reports

There were no adverse events reported by concerned parents, teachers, or project coordinators.

## Discussion

To our knowledge, this is the first and the largest trial assessing the intervention effectiveness of obesity prevention on blood pressure in children and adolescents. This average 6-month school-based, family-involved, comprehensive intervention seems not to affect both weight and blood pressure in all the children and adolescents aged 6–18 years being studied. However, subgroup analyses revealed that children aged 6–12 years were more sensitive and the intervention on preventing overweight and obesity and HBP more effective than in those aged 13–18 years.

Given the significant downward trend of blood pressure in younger children during the intervention period, it is plausible that the comprehensive obesity-targeted intervention in China has an obvious effect on decreasing the HBP risks in children aged 6–12 years, especially in those with higher BMI levels. Previous studies and pediatric hypertension guidelines had emphasized the importance of comprehensive prevention and intervention strategies on blood pressure, including lifestyle intervention and the control of obesity so as to maintain a healthy blood pressure level in children (2, 19). However, evidence has been lacking in the pediatrics clinical practice or epidemiological studies. Our findings in the multicenter non-randomized controlled trials (RCTs) demonstrated that the comprehensive school-based obesity intervention, which started in early childhood and particularly in those with overweight status, might be effectively used to decrease both obesity and HBP risks.

Previous reviews of childhood obesity prevention focused largely on schools were numerous, and the findings have been inconsistent. One meta-analysis found that, of the 147 RCTs conducted to prevent or treat childhood obesity in high-income countries, few have demonstrated successful BMI change, but with small effect sizes (20). One latest RCT conducted in United States also found that a 36-month behavioral intervention did not change the BMI trajectory among underserved preschool-aged children (21). The RCT conducted in the UK with an intervention of 24 months also reached an insistent conclusion that no effect was found for the intervention on preventing overweight or obesity (10). However, some other studies provided evidence on the effectiveness of obesity intervention. One RCT conducted in Sweden found that comprehensive obesity interventions based on parental support program to promote healthy dietary and physical activity habits showed significant intervention effects (22). Consistent conclusions were also reported in other studies (7, 23). However, different settings, a single intervention measure, or the sample sizes may limit scaling up the research. Schools have been identified as a recommended setting for public health strategies to preventing childhood obesity (8). Our studies added the evidence that a comprehensive intervention in the school setting with family involvement is more effective in preventing overweight and obesity in younger children who are the priority of intervention strategies. The development of precise intervention measures and non-standardized or general intervention programs is warranted for policy-making in the future.

Theory and evidence are abundant in the literature about HBP being positively associated with overweight and obesity. In addition, lifestyle factors, including sedentary behaviors, physical activity, unhealthy dietary habits, and adverse family environment, are noted as the major contributing factors to HBP in children and adolescents (24). In the specific group, our findings confirmed the necessity of a school-based comprehensive obesity intervention implementation combined with lifestyle factors for improvements in children's blood pressure levels and HBP risks. One previous meta-analysis of 25 trials showed reductions in SBP and DBP levels due to weight loss during the intervention period in adults (25). Feasible biologic pathways may address the effects of weight intervention on the decreased risk of HBP in children. The renin–angiotensin–aldosterone system is overactivated early in obese children, whose renin activity and aldosterone concentrations are higher than those in lean peers (26, 27). But the sympathetic nervous system is more active in hypertensive and obese subjects (28). Furthermore, weight interventions may inhibit the natriuretic peptide system, of which the functional effects are vasodilatation and natriuresis (29). In addition, decreased insulin sensitivity and hyperinsulinemia due to weight loss and lifestyle factor reduction might also provide evidence for the decline of hypertension risks (30). More obvious effects in children with higher BMI percentiles, also in the present study, confirmed the biological pathways above, a shift from an epidemiological perspective.

An interesting finding of the present study is that there was a significant age difference in the effective evaluation of the obesity intervention in that children aged 6–12 years showed more sensitivity to the intervention, but not those in middle and late adolescence (aged 13–18 years). Childhood and adolescence are two different but closely linked periods before adulthood. Adolescence is a critical transition period between childhood and adulthood, which brings rapid physical growth and behavioral alterations. However, girls begin reaching puberty with an average age of menarche at about 12 years, although with a 2-year lag in boys (31). After the onset of puberty, secretions of different hormones begin to accelerate, including follicle-stimulating hormone, luteinizing hormone, testosterone, and estradiol levels (32, 33). It has been proven that an obvious increase in sex hormone secretion has an important influence on blood pressure regulation (34), whose effects might be more pronounced in middle and late adolescence. In addition, studies have shown that obesity is associated with earlier puberty onset (35, 36). In addition, it is hard to change some behaviors in adolescents compared to their younger peers (37). Thus, obesity intervention in childhood, before 12 years, targeting some key factors, such as lifestyle factors and environmental changes, could produce more effective effects on the blood pressure levels and HBP risks. One small pilot trial conducted in Germany also found that early preventive measurements in overweight and obese primary school children were effective in decreasing blood pressure levels (38). Although no significant effects were found in adolescence, more declines in the BP levels and HBP risks, and even in the significant DBP *Z*-scores, in adolescents aged 16–18 years after the intervention in the intervention group compared to the control group reflected that the significance of the comprehensive intervention in adolescents may not be apparent temporarily due to a few crucial factors such as the intensity and duration of the intervention measures. Thus, intensive or precise interventions in adolescents could have a positive effect. One study reported the effective effects of a school-based obesity intervention on BP levels only in adolescents (11). They were different from the findings of our study, but provided evidence of the meaning of continued intervention for adolescents. Recent studies confirmed that weight increments in early childhood had a higher risk of cardiovascular disease than those overweight or obese individuals in adolescence (39). Thus, our study not only confirmed the importance of early obesity intervention for HBP prevention in childhood but also provided directions for future interventions: that preventing HBP in adolescence may require more efforts and larger changes in weight. Given the importance and difficulty of interventions in adolescence, precise interventions, even costly, need to be developed further.

Previous studies reported that the risk of HBP would increase for individuals whose body status changed from normal weight to overweight and obesity during a long period, but they failed to examine the effects of weight loss on preventing HBP in adults (40, 41). Weight changes were important for the control of hypertension in children and adolescents during and after the intervention period (42). Our analyses found that the significant intervention effects in children aged 6–9 years disappeared after stratification according to the weight changes before and after intervention, with undifferentiated HBP prevalence between the control group and the intervention group. This finding provided evidence that weight changes during the intervention period might mediate the effects of intervention on the blood pressure levels and HBP risks. We also found inconsistent trends of the HBP difference in the two age groups, with declined trends in children aged 6–12 but increased trends in adolescents aged 13–18 years in the increased weight group pre- and post-intervention period, while without statistically significant prevalence gaps. However, the decrement for HBP prevalence in children aged 6–12 years was larger and the increment in adolescents aged 13–18 years was smaller for individuals whose weight had increased in the intervention group than for those in the control group, which also occurred in both children and adolescents whose weights declined or remained stable. Compared to children with lower BMI percentiles at baseline, those with higher BMI percentiles had more obvious intervention effects for HBP prevention, which reflected that children with higher body weight may be more responsive to comprehensive interventions in the short term. Previous studies found that the baseline BMI is associated with a future incident risk of hypertension, even after accounting for weight change during the follow-up period; thus, the impact of obesity on incident hypertension was independent of weight gain among adults (41). The average BMI used in many other studies was not a complete reflection in predicting the BP changes because it counteracted the difference in the different percentiles, might be in the bottom two, but the changes in BP with BMI percentiles were not even. Therefore, this finding reinforced evidence that sustained weight intervention and management as early as possible in childhood, and throughout adolescence, is effective and necessary in preventing HBP risks.

The present study is the first and largest multicenter school-based and family-involved obesity intervention and BP effectiveness assessment study in Chinese children and adolescents. Although the selection of schools in each center did not realize the randomization, willingly participating schools will increase the degree of cooperation for intervention and the effectiveness of the implementation program. Thus, the design of the multicenter cluster non-randomized controlled trial provided a strong reference for obesity intervention on HBP prevention in Chinese children and adolescents with a broad age band and a large sample size, especially valuable for the present weak to null results among adolescents. However, the undesirable intervention results produced in those older might suggest that we should not waste resources in a general intervention among all adolescents in the future. The more precise, the more helpful; in other words, adolescents could not obtain more benefits from a general intervention, but more need of a precise intervention because the target population among them is small. In addition, the primary outcomes of the present study were fully assessed using a comprehensive set of variables around the theme of weight and blood pressure in order to capture potential intervention effects on later cardiovascular risks, including weight and blood pressure levels and their standardized (including BMI, SBP, and DBP *Z*-scores) and risk (including overweight and obesity, SHBP, DHBP, and HBP) outcomes.

However, three limitations should be mentioned. Firstly, because the study was conducted among Chinese children and adolescents, the findings might not be generalized to other populations. However, a lot of previous literatures were conducted in high-income countries, so our study should provide a good reference for middle- and low-income countries. Secondly, the present results adopted the classical BP criteria developed in American children in 2004 (18), and we did not use the updated US BP criteria in children (19), but the 2004 criteria were widely applied by researchers, which makes it easy to directly compare our results to those of other studies. The latest studies have demonstrated that the associations between HBP with BMI and other medical and behavioral factors remained unchanged using the updated US BP criteria, so our results would not be affected by the different BP definitions. Thirdly, data on lifestyle factor changes, such as dietary behaviors, physical activity, health literacy, and family support, during the follow-up period and their independent effects on obesity and HBP risks were not analyzed, which need future research.

## Conclusion

In summary, a 6-month multicenter school-based and family-involved obesity intervention in China did not observe overall risk reductions of overweight and obesity and HBP in children and adolescents aged 6–18 years. However, children aged 6–12 years could be more sensitive to the intervention, with a more significant drop in overweight and obesity and in HBP risk than those aged 13–18 years. The intervention in children aged 6–12 years with overweight status can yield a more effective blood pressure control. Therefore, a comprehensive health lifestyle intervention was needed as early as possible in school settings in order to reduce the risks of obesity, HBP, and other cardiovascular diseases in the future. Future research with longer-period precise interventions should assess the effectiveness of interventions in adolescents.

## Data Availability Statement

The raw data supporting the conclusions of this article will be made available by the authors, without undue reservation. Proposals should be directed to majunt@bjmu.edu.cn and songyi@bjmu.edu.cn.

## Ethics Statement

This project was approved by the Medical Research Ethics Committee of Peking University Health Science Center (IRB00001052-13034). Written informed consent to participate in this study was provided by the participants' legal guardian/next of kin.

## Author Contributions

YD conceptualized and designed the study, completed the statistical analyses, drafted the initial manuscript, and reviewed and revised the manuscript. YS, YM, and JM contributed to the conceptualization and design of the study, supervised the data collection, statistical analyses, initial drafting of the manuscript, and reviewed and revised the manuscript. ZZ and HW assisted with the statistical analyses and critically reviewed and revised the manuscript. PH and BD assisted with the data processing, statistical analyses, and the interpretation of the data. All authors approved the final manuscript as submitted and agreed to be accountable for all aspects of the work.

## Conflict of Interest

The authors declare that the research was conducted in the absence of any commercial or financial relationships that could be construed as a potential conflict of interest.
