# Supplementary material for: National School-Based Health Lifestyles Intervention in Chinese Children and Adolescents on Obesity and Hypertension
Source: Front Pediatr. 2021 May 28;9:615283. doi: 10.3389/fped.2021.615283 (PMC8192970; doi:10.3389/fped.2021.615283)
Supplement: Supplementary file 1 [file Table_1.DOCX]

Supplementary Material


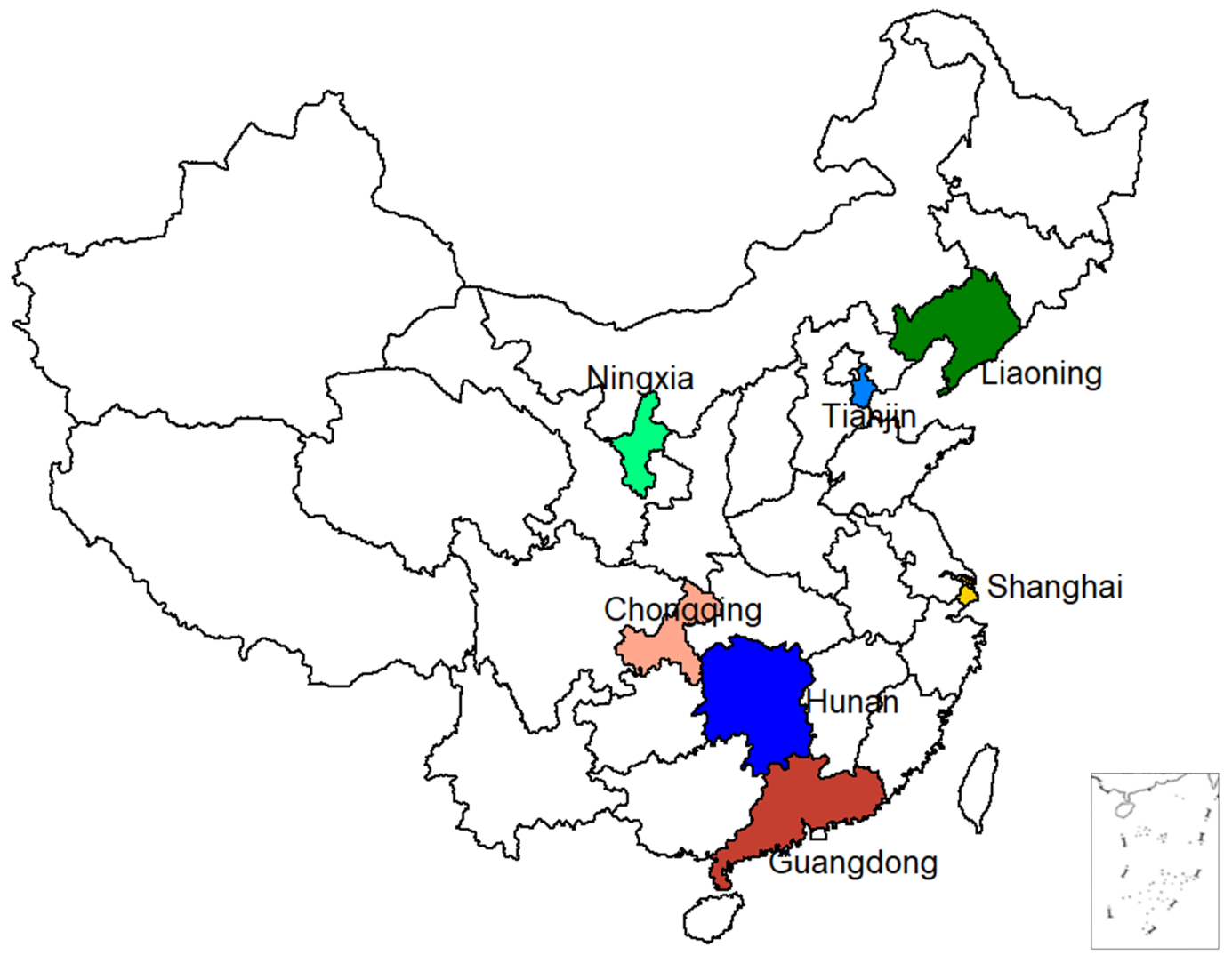


**Figure S1.** The geographic distribution of involved provinces and municipalities

Note: This national multi-centered school-based cluster nonrandomized controlled trial Health Lifestyles Intervention in Chinese Children and Adolescents (HLI-CCA) was conducted in seven provinces and municipalities including Liaoning, Ningxia, Shanghai, Chongqing, Hunan, Tianjin, and Guangdong.


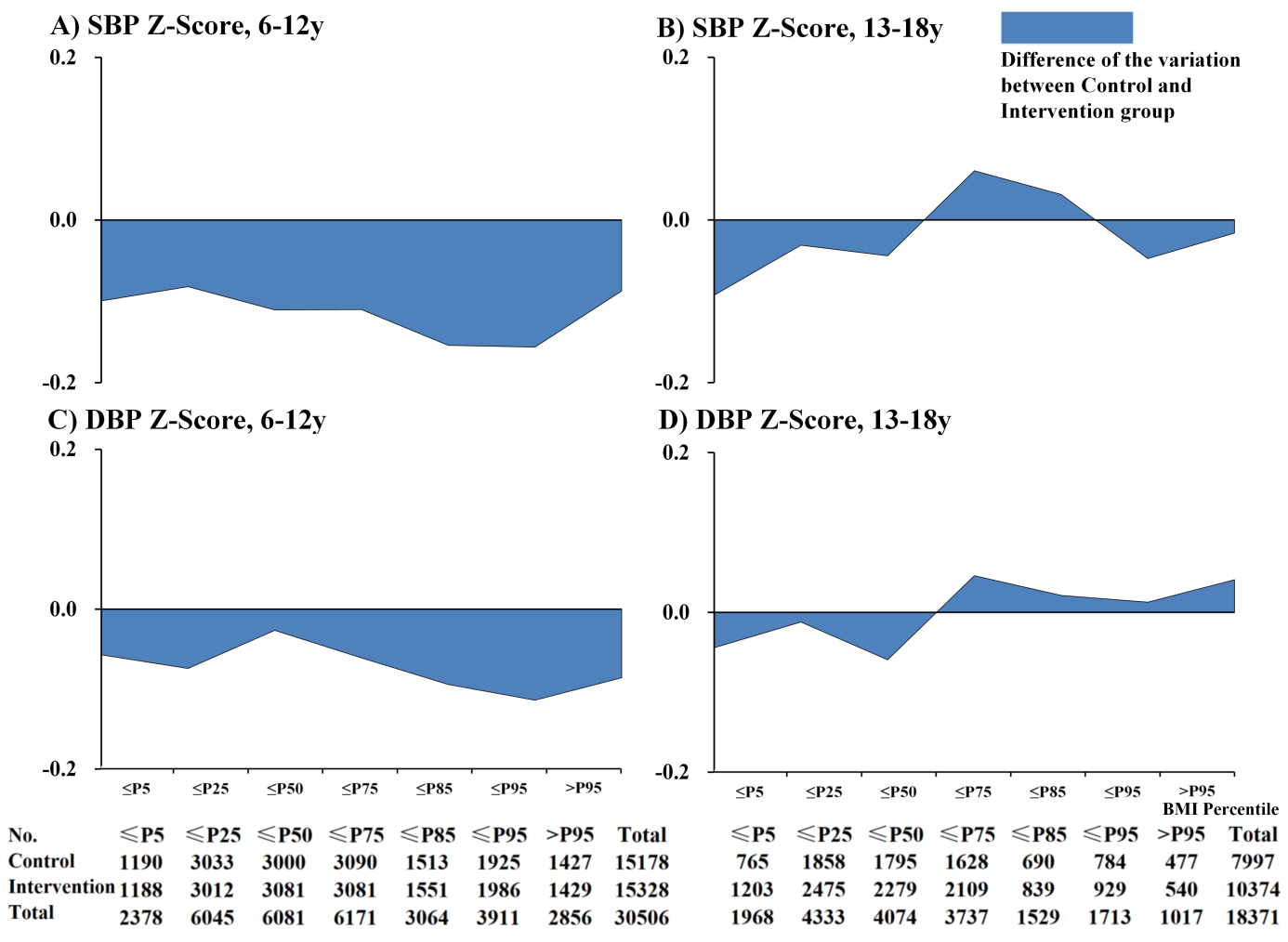


**Figure S2.** Comparison of change values of systolic blood pressure (SBP) and diastolic blood pressure (DBP) standardized values during the period of prior- and post-intervention between Control and Intervention group in different BMI percentiles at baseline in two age groups of 6-12 years and 13-18 years

Note: Blue areas represented the change values of variation between Control and Intervention group during the period of prior- and post-intervention. Blue areas under the zero lines represented that the decrement in Intervention group was higher than that in Control group or increment in Intervention group was lower than that in Control group. In each BMI percentiles group, change values= (Standardized values Z-Score in post-intervention in Intervention group- Standardized values Z-Score at baseline in Intervention group) - (Standardized values Z-Score in post-intervention in Control group- Standardized values Z-Score at baseline in Control group). That is, blue areas under the zero lines represented effective intervention effects, but above the zero lines represented invalid intervention effects.
